# Mining co-location patterns of manufacturing firms using Q statistic and additive color mixing

**DOI:** 10.1371/journal.pone.0299046

**Published:** 2024-03-06

**Authors:** Yi Song, Guanglei Li, Yihan Wang, Yiheng Wang, Chang Ren

**Affiliations:** 1 Key Laboratory of Urban Land Resources Monitoring and Simulation, Ministry of Natural Resources, Shenzhen, Guangdong, China; 2 Development Research Center for Natural Resource and Real Estate Assessment, Shenzhen, Guangdong, China; 3 School of Urban Planning and Design, Peking University, Shenzhen, Guangdong, China; 4 School of Resource and Environmental Sciences, Wuhan University, Wuhan, Hubei, China; 5 Research Center for Eco-Environmental Sciences, Chinese Academy of Sciences, Beijing, China; 6 College of Air Traffic Management, Civil Aviation Flight University of China, Guanghan, Sichuan, China; Libyan Academy, LIBYA

## Abstract

The agglomeration effect significantly influences firms’ site selection. Manufacturing firms often exhibit intricate spatial co-location patterns that are indicative of agglomerations due to their reliance on material input and product output across various subdivisions of manufacture. In this study, we present an analytical approach employing the Q statistic and additive color mixing visualization to assess co-location patterns of manufacturing firms. We identified frequent pairs and triplets of manufacturing divisions, mapping them to reveal distinct categories: labor-intensive clusters, upstream/downstream industrial chains, and technology-spillover clusters. These agglomeration categories concentrate in different regions of the city. Policy implications are proposed to promote the upgrade of labor-intensive divisions, enhance the operational efficiency of upstream/downstream industrial chains, and reinforce the spillover effects of technology-intensive divisions.

## Introduction

As a primary determinant of industrial location [1], the agglomeration effect refers to the advantage firms gain from close proximity to each other. Since the 1990s, industrial agglomeration has garnered significant attention, especially with the emergence of the new economic geography. Collaborative efforts between governments, public and private enterprises [2], and other entities have led to the establishment of various industrial agglomerations or clusters [3], aiming to capitalize on resources in production and demand [4]—including infrastructure, market access, labor force, and industrial chains [5, 6].

To optimize industrial layout, scholars have explored the characteristics and patterns of industrial agglomeration, investigating the driving factors and determinants through Marshall’s externality theory [7], Weber’s industrial location theory [8], and Krugman’s new economic geography [9, 10]. Empirical quantitative studies have emerged to assess the suitability and uncertainty of these theories in various cases. Using aggregated census data, researchers measured industry concentration in specific regions through indicators such as the Gini index [11], Herfindahl-Hirschman index [12], Ellison Glaeser index [13], and Theil index [14]. However, assuming interior homogeneity within a region, these studies offer insights limited to the entire region, failing to extrapolate to specific clustering patterns within local areas and individual enterprises. This introduces the risk of ecological fallacy. Importantly, adjusting the size of geographic measurement units doesn’t fundamentally address this limitation; rather, it introduces variations in results, presenting the challenge of the multiple areal unit problem [15, 16].

To understand the regional agglomeration of industries, it is beneficial to analyze the location pattern of firms within a region at a local scale, shifting perspectives from regions as the theater to firms as the actor. For instance, Arku et al. [17] developed policy implications for the spatial configuration of Ontario firms across 26 manufacturing divisions based on point density analysis. Albert et al. [18] revealed the siting pattern of Spanish manufacturers by measuring the geographical concentration of manufacturing departments at multiple scales using Ripley’s K function. Moreno-Monroy et al. [19] analyzed the clustering of Colombian manufacturing firms using M function and kernel density mapping. Garrocho-Rangel et al. [15] compared planar and network K-functions in the analysis of the localization of tertiary firms in the Central Business District (CBD) of metropolitan Toluca, Mexico, demonstrating the advantages of the planar approach. However, these studies primarily focus on examining the general spatial arrangement of firms within the same sector, overlooking potentially intricate interconnections between different sectors. This disregards the significance of cross-sectoral agglomeration, as highlighted by Jacobs’ externalities [20]. From a geographical perspective, interactions between neighboring geographic features should be considered [21], in addition to spatial distribution. Therefore, there exists a research gap in the lack of analysis on the interactions between firms, especially after distinguishing the specific sectors to which they belong.

The manufacturing industry constitutes a significant source of tax revenue and employment in China’s economy. However, it is currently undergoing an industrial transformation [22], encountering challenges related to industrial upgrading in relevant industrial zones. The spatial layout of the manufacturing industry can be optimized and intervened [23] to promote the sustainable development of regions or industrial zones by analyzing the agglomeration pattern of manufacturing firms [24]. Interactions between these firms are complex, with multiple divisions playing different roles in the industrial chain, often shaped by intricate supply and value chains covering multiple sectors. With the advent of a new technological revolution and profound industrial changes, these interactions are increasingly pronounced, exhibiting spatial explicitness and geographical measurability. Addressing the identified gap becomes even more critical for the manufacturing industry, gaining demonstrative significance. The research question of this study is formulated as “How to grasp the pattern of regional manufacturing industrial agglomeration by capturing the intricate interactions among numerous firms across multiple sectors?”

The combination of spatial event types frequently occurring in the same region can be identified through co-location pattern mining, using various techniques from spatial statistics and data mining [25, 26]. Applications in urban industry clusters and functional zoning have been reported [27, 28]. Since the framework of spatial co-location pattern mining was founded [25, 29], this topic has received continued concern to address several key issues, including scalability and statistical performance, adaptation for network and flow data, and interpretation of identified prevalent patterns. Zhang et al. [30] improved the spatial join algorithm to allow for fast analysis of long patterns. Huang et al. [31] and Yu et al. [32] adapted the methods for rare events and distance-decay effects. Wang et al. [33] and Yang et al. [34] developed variants for accelerated search and parallel computation. Tian et al. [35] and Cai and Kwan [36] exemplified the co-location analysis in network space and among origin-destination pairs. Deng et al. [37] proposed a multi-level approach for effective identification of local patterns. Wang et al. [38] attempted to address the problem of repetitive counting during the calculation of metrics. Anselin and Li [39] explored the spatial pattern of significant clusters at the local level with a full observation of the population. Liu et al. [40] emphasized the statistical significance of identified co-location patterns.

While kernel density estimation of the distance distribution [26], K function [41, 42], and the colocation quotient [43, 44] are noteworthy strands of study, the analysis of colocation patterns has been limited to autocorrelation among a set of points or the correlation between two sets of points. For the analysis of points with multiple types, these approaches can be applied multiple times to all possible pairs of types. In the case of industrial or manufacturing chains, there are potentially longer patterns of colocation involving triplets or even more types to be considered as a single pattern. Among other studies, Sierra and Stephens [45] represented the identified co-location patterns using a network-based visualization technique, acknowledging the difficulty in the interpretation of mining results. Yang et al. [46] also argued that the coupling relation was important to make sense of the co-location patterns.

Q test detects spatial association patterns in categorical variables, based on the Q statistic of spatial independence, featuring desirable statistical inference capability for co-location pattern mining. It supports the analysis of variables with multiple categories, which is still favorable. The theoretical foundation of this test is symbolic dynamics [47], and the test has been applied in various analysis scenarios, such as the spatial association of fast-food restaurants in Toronto, ethnicity clustering of residents in Newark [48], comparison of thematic maps [49], and co-location pattern mining of hotels [50]. Therefore, it would be valuable if the method could be scaled to the analysis of large datasets with multiple categories, and prevalent co-location patterns from its output could be effectively analyzed to make sense of the spatial patterns and processes. The additive color mixing technique [51] is a method to map and analyze spatial distributions of geographical phenomena through pseudo color composites and visual thinking. It provides an intuitive, comprehensible, and perceivable approach to analyze spatial patterns.

This study aims to identify co-location patterns from large datasets, including potential co-located triplets or even longer patterns, in addition to pairs that have been the focus of previous studies. We propose a creative approach to co-location pattern analysis based on Q-statistics and additive color mixing visualization. This approach is subsequently applied to the empirical study of a specific region distributed with a large number of manufacturing firms. The adaptation of the Q statistic using k-dimensional tree (kd-tree) enables the detection of colocation patterns for large datasets. The identified patterns are interpreted based on additive color mixing visualization to facilitate an intuitive and impressive presentation of the patterns. Therefore, this study demonstrates a practical solution using both classical statistical and novel visual techniques to explore the co-location patterns of large datasets, contributing to the knowledge base of the effective adaptation and application of analytic tools for understanding industrial agglomeration patterns.

## Method

In this study, we utilized the Q statistic to extract frequent co-location patterns from the addresses of manufacturing firms. Given the substantial number of such firms, we adapted the calculation of the Q statistic using kd-tree to reduce the memory requirement. Due to the challenge in interpreting [28] identified co-location patterns of multiple categories, i.e., manufacturing divisions, we employed the visualization technique of additive color mixing to enhance the understanding of both the location and the pattern of co-located firms. Our goal was to visualize the mixing and spatial patterns where some combinations of firm types are more prominent or more densely distributed at specific parts of the city.

### Q statistic with adaptation for large datasets

**Fig 1:**
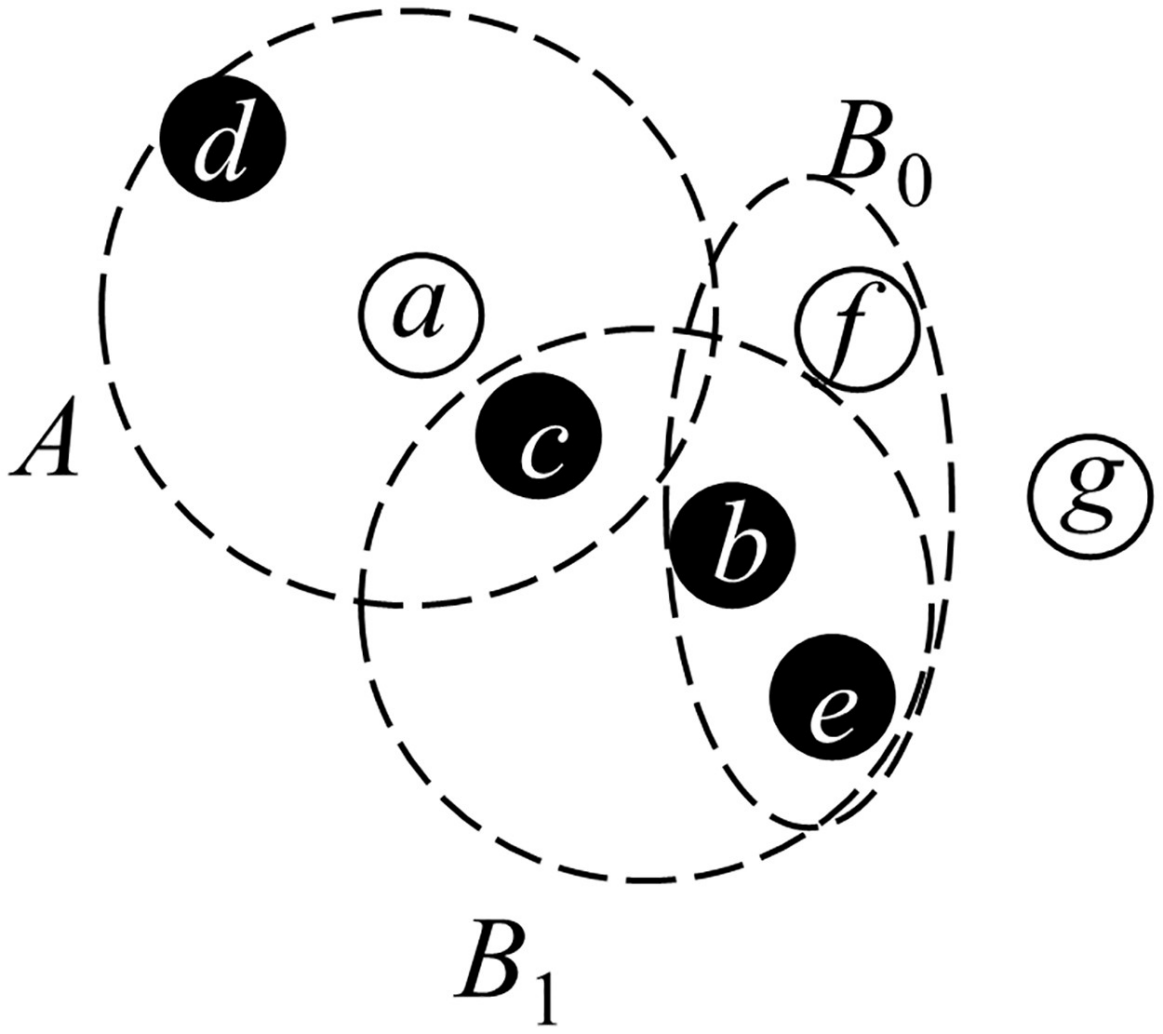
An example of *m*-surrounding and overlapping degree.

The Q statistic [47] is defined based on the frequency distribution of the symbolized representation of a point feature and its *m* − 1 nearest neighbors (referred to as *m*-surroundings). Two types of symbolized representations exist for *m*-surroundings, which are a sequence of point types within the *m*-surroundings. A representation is termed a standard symbol if the sequence is ordered by distance from the point, while the equivalent symbol is a representation of an unordered set of types. To mitigate symbol dependency caused by overlapping *m*-surroundings of nearby points, it is essential to restrict the degree of overlap by sampling the population of *m*-surroundings. The sampled symbolized representation is referred to as symbolized observations. The maximum number of symbolized observations can be determined by the size of surroundings *m* and the overlapping degree *r*. For instance, in Fig 1, points *c* and *d* are the two nearest points from *a*, forming the triplet *A* (comprising *a*, *c*, and *d*) as the 3-surrounding of point *a*, as shown in the figure. Then, we aim to identify the 3-surrounding of point *b*. Although *e* and *c* are the two nearest points from *b*, point *c* has already been included as part of the triplet *A* in the 3-surrounding of point *a*. If the overlapping degree *r* is one, point *c* is allowed to be part of the triplet *B*_1_ as the 3-surrounding of point *b*. However, if *r* is zero, signifying no overlapping is allowed, the 3-surrounding of *b* must include the third nearest point *f*, forming the triplet *B*_0_ of points *b*, *e*, and *f* as the 3-surrounding of point *b*. Recall the definition mentioned at the beginning of this subsection; for a 3-surrounding, if the order is considered, we have *white*, *black*, *black* as the standard symbol of *A*(*a*, *c*, *d*). Otherwise, the equivalent symbol would neglect the order of distance and consider all triplets with 2*black*, 1*white* as the same pattern. Then *B*_0_(*b*, *e*, *f*) would be another instance of this pattern of the equivalent symbol, although its standard symbol *black*, *black*, *white* is different.

The frequency of symbolized observations can be utilized to calculate the entropy of the sampled spatial process. Q statistics can then be defined based on the upper bound and observed entropy, following a chi-squared distribution with a degree of freedom of *k*^*m*^ − 1 (*k* is the number of possible point types). For mathematical details, refer to the S1 Appendix. Therefore, for groups of point features of different types, this statistic can be employed to test if the spatial distribution of point types is random. If it is not, a co-location pattern exists.

The calculation of Q statistic involves the construction of *m*-surroundings. The implementation of the algorithm in the existing R software package creates a distance matrix of *N* × *N*. When building the *m*-surroundings iteratively, the processed items need to be flagged to control the degree of overlap during the sampling process. In the R implementation, rows and columns corresponding to the flagged items are deleted from the distance matrix. However, with a large amount of data, constructing the distance matrix of *N* × *N* becomes impractical. In this study, for instance, the number *N* of samples reached 190,612, and an ordinary computer could not handle the construction of such a large-scale distance matrix.

**Fig 2:**
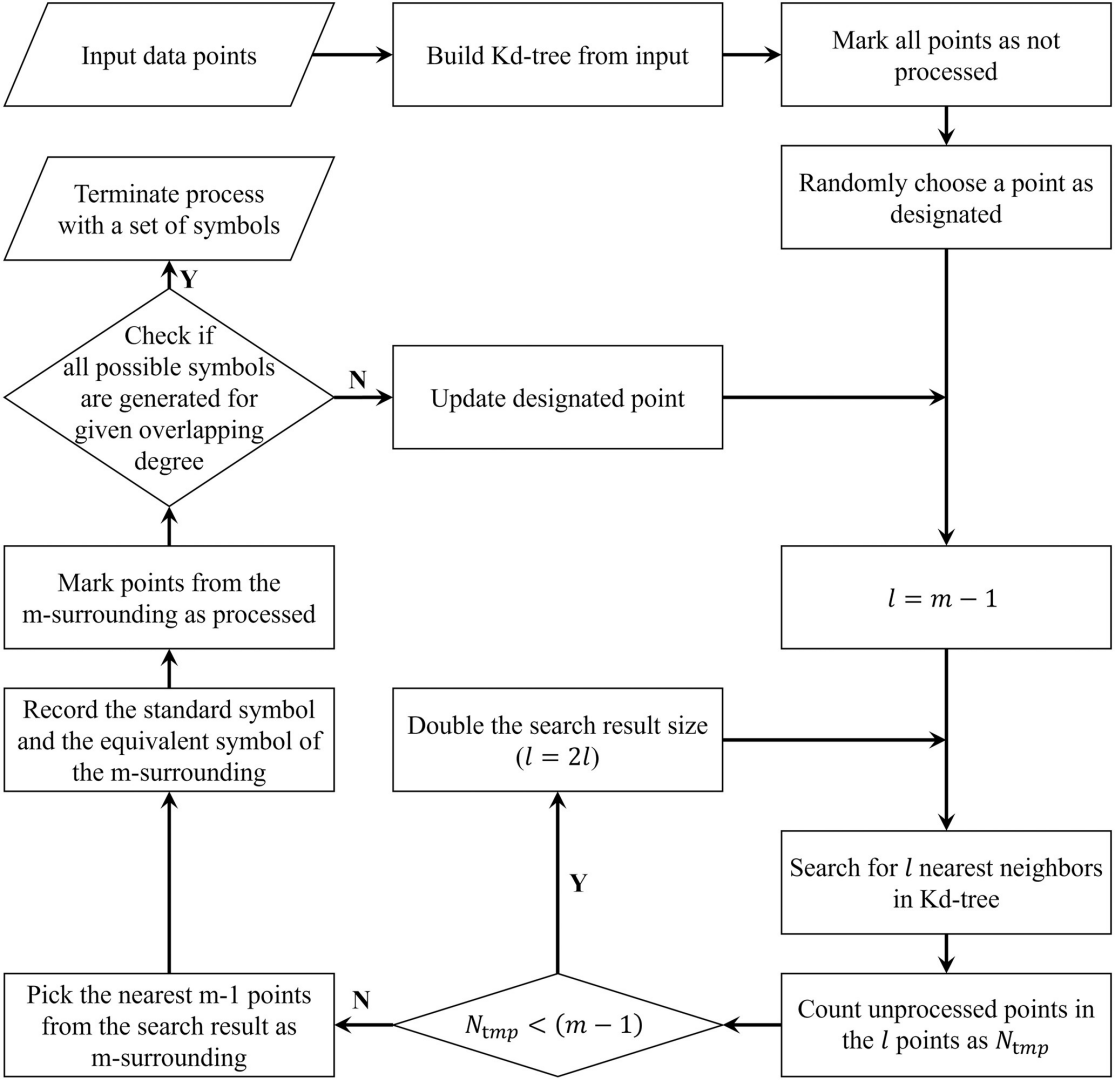
Flow chart of the Q statistic calculation for large datasets.

Therefore, the distance matrix was not directly calculated in our study to reduce space and time complexity (Fig 2). Instead, a spatial index was built using the K-dimensional tree (Kd-tree) algorithm [52]. This algorithm recursively splits the *n* points in K-dimensional space into binary trees, dividing *n* − *K* datasets. The Kd-tree spatial index was used to search the neighborhood for a fixed number of nearest points, which were then flagged as processed to be skipped in further steps. To find *m* − 1 unflagged points nearest to the designated point in each iteration, a nearest neighbor point search scheme with variable step sizes was adopted. First, *l* (*l* = *m* − 1) points nearest to the specified point were searched. If there were any processed points, the search was conducted again within a larger extent of 2*l* points, until the number of unflagged points from this search was not less than *m* − 1. The *m*-surrounding of other unprocessed points was then constructed according to the algorithm. After this improvement, the calculation of Q statistic for a relatively large amount of data became feasible (S3 Appendix).

### Visual analytics of colocation pattern using additive color mixing

To illustrate the geographical distribution of significant co-location patterns detected and tested by the Q statistic, we aimed to map the spatial locations of noteworthy co-location patterns among firms across various manufacturing sectors. This approach was inspired by the visual mining method based on the law of additive color mixing for representing interrelated spatial phenomena in different visual depths [51].

The fundamental concept of this technique involves generating a pseudo-color image through density estimation of spatial point patterns. The density of each spatial point pattern is then mapped to the intensity of a color space, such as red-green-blue (RGB). Consequently, the hue of a pixel in this image represents a unique mixing pattern of spatial points. Throughout this process, the density of each phenomenon is linearly rescaled to the range of 0 to 255 for an 8-bit image. This representation indicates the relative density within a certain type of point phenomenon and is not comparable across types. There is also a limitation on the number of types due to the three-dimensionality of the color space, allowing visualization of only three or fewer types of spatial point patterns. Nonetheless, this restriction aligns with the study’s design, grouping manufacturing sectors based on the property of firms into labor-intensive, intermediate-demand, or technology-intensive ones.

For a significant co-location pattern represented as a triplet of sectors, we mapped the supporting instances of firms based on their addresses to explore the specific locations where this pattern occurs. The density of each sector within the triplet of co-located sectors was mapped to the intensity of a color channel and mixed additively to determine the color of each pixel in the map. Utilizing this additively mixed pseudo-color map image, we conducted further analysis on the most significant co-location patterns.

## Data and preprocessing

Shenzhen is situated on the east bank of the Pearl River Estuary, in the southern part of Guangdong Province, China. As a megacity, it covers a total area of about 1997 square kilometers. Shenzhen City holds a prominent position in the industrial division of manufacturing communication, computer, and other electronic equipment in China. Meanwhile, Guangdong Province leads in the industrial division of manufacturing chemicals and chemical products, metals, transportation equipment, and electrical machinery and equipment. Labor-intensive industries like the manufacturing of textiles, wearing apparels, and furniture continue to attract a significant workforce. Therefore, the spatial layout of manufacturing firms in Shenzhen serves as a demonstrative case for our proposed approach.

**Fig 3:**
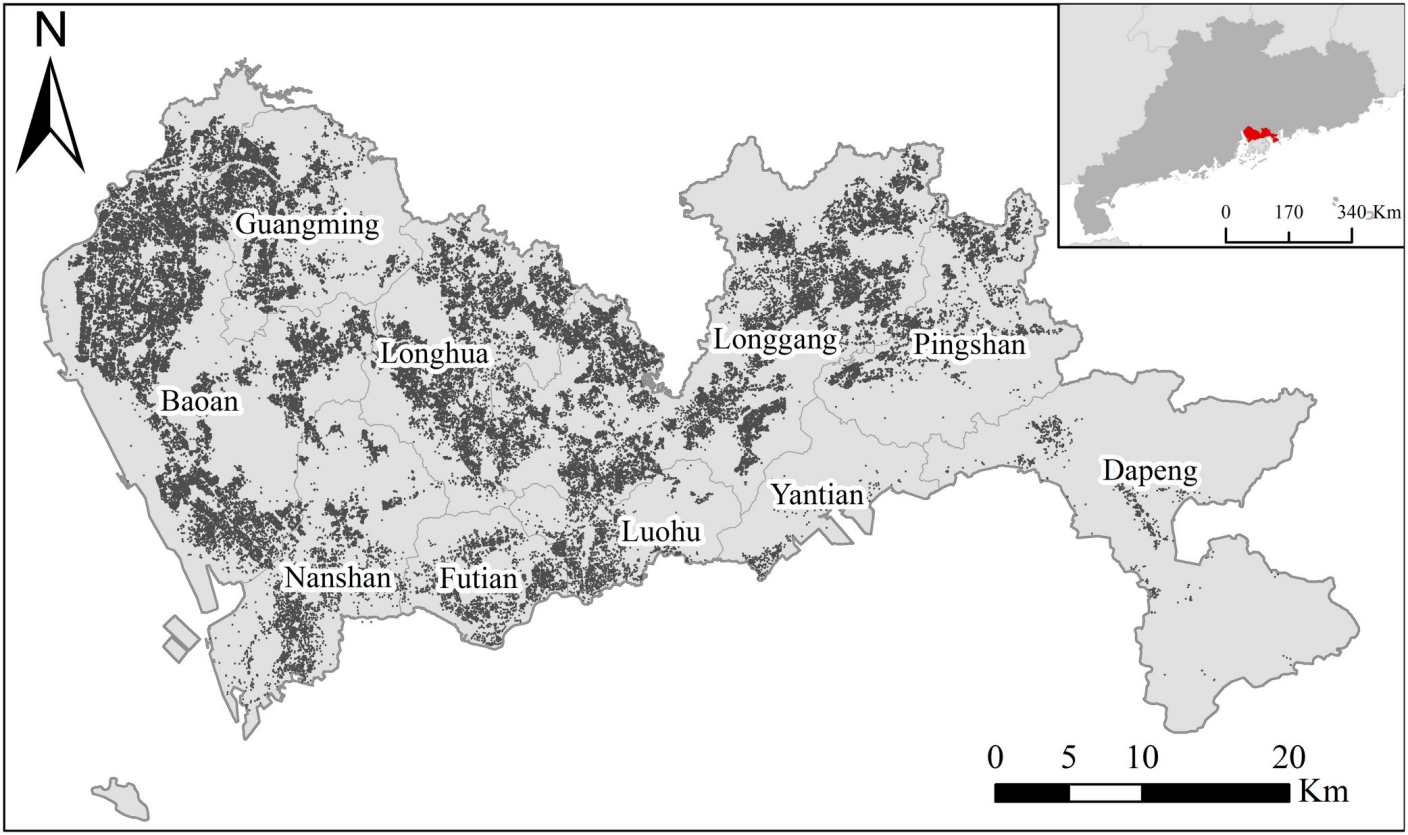
Manufacturing firms in Shenzhen. (Source: The authors).

The original data utilized for this study included the business status and registered spatial locations of firms in Shenzhen in 2018. These recorded firms spanned 20 sections, 96 divisions, 532 groups, and 1405 classes of industries according to the Chinese taxonomy. The dataset underwent cleaning and validation, involving the removal of records with missing or invalid attributes and the elimination of duplicate entries. Following preprocessing, 2.86 million entries of valid data were obtained. Specifically, a total of 190,612 entries of data for the manufacturing industry (classified under section code “C”) were extracted for geocoding and used in this study. A map depicting these manufacturing firms in Shenzhen is presented in Fig 3.

The manufacturing industry in Shenzhen is dispersed throughout the city, excluding Dapeng, Pingshan, and Yantian. For the purpose of this study, we group the manufacturing of wearing apparels (C18) and educational and sports items (C24) under the label of labor-intensive divisions. The manufacturing of metal products (C33) and rubber and plastic products (C29) is categorized as intermediate-demand divisions, while the manufacturing of computers (C39), electrical machinery (C38), and special-purpose equipment (C35) falls under the technology-intensive divisions.

## Results and analysis

### Q tests and prominent co-location patterns

For this study of manufacturing firms in Shenzhen, *N* = 190,612, and the categories of manufacturing firms (*k* = 31) are presented in Table 1.

**Table 1:** Percentages of manufacturing firms by industrial division.

Industrial division	Percentage
C13–Processing of farm produce	0.25%
C14–Manufacture of food products	0.69%
C15–Manufacture of beverages	0.11%
C16–Manufacture of tobacco products	0.02%
C17–Manufacture of textiles	0.89%
C18–Manufacture of wearing apparels (except fur apparels)	11.21%
C19–Manufacture of leather, fur, feather products and footwear	2.55%
C20–Wood processing and manufacture of wood, bamboo, and plaiting products	0.53%
C21–Manufacture of furniture	1.94%
C22–Manufacture of paper and paper products	2.68%
C23–Printing and reproduction of recording media	1.76%
C24–Manufacture of educational, artistic, sports and recreational items	3.81%
C25–Manufacture of coke, refined petroleum products, and nuclear fuel	0.05%
C26–Manufacture of chemicals and chemical products	1.09%
C27–Manufacture of pharmaceuticals	0.61%
C28–Manufacture of chemical fibers	0.07%
C29–Manufacture of rubber and plastic products	7.23%
C30–Manufacture of non-metallic mineral products	1.17%
C31–Ferrous metal smelting and rolling processing	0.29%
C32–Non-ferrous metal smelting and rolling processing	0.27%
C33–Manufacture of fabricated metal products	10.77%
C34–Manufacture of general-purpose machinery and equipment	2.53%
C35–Manufacture of special-purpose machinery and equipment	3.68%
C36–Manufacture of automobiles	0.30%
C37–Manufacture of for railway, water, aerospace and other transport equipment	0.30%
C38–Manufacture of electrical machinery and equipment	6.47%
C39–Manufacture of computer, communication and other electronic equipment	26.19%
C40–Manufacture of instruments	1.89%
C41–Other manufacturing	10.29%
C42–Comprehensive utilization of waste resources	0.16%
C43–Repair of metal products, machinery and equipment	0.20%

The size of the *m*-surrounding depends on the number of points *N*, possible number of types *k*, and the overlapping degree *r*. If the *m*-surrounding is too large, there will not be enough samples to justify the test (Paez et al., 2012). As a requirement of statistical power, the test needs at least 5*k*^*m*^ symbolized samples, i.e., *N* > 5*k*^*m*^, and thus $m<\frac{\text{ln}(N/5)}{\text{ln}\;k}$. Hence, the size *m* in this study should be less than 3.07. While this parameter means the number of points in proximity, it has to be an integer and at least 2 to form a neighborhood. Therefore, the value of parameter *m* can only be 2 or 3. Three groups of experimental parameters ((*m*, *r*) = (2,0), (2,1), (3,2)) were used in this study, as shown in the heading of Table 2.

**Table 2:** Parameter settings and results for Q tests.

Parameter or statistic	Value		
	Group 1 *m* = 2, *r* = 0	Group 2 *m* = 2, *r* = 1	Group 3 *m* = 3, *r* = 2
Number of cases *N*	190612	190612	190612
Symbolized observations *R*	95306	190611	190610
Number of classes *k*	31	31	31
Size of *m*-surrounding *m*	2	2	3
Max size of *m*-surrounding *m*_*Max*_	3	3	3
Overlap degree *r*	0	1	2
Number of standard symbols *k*^*m*^	961	961	29791
Number of equivalent symbols *k*(*k* + 1)…(*k* + *m* − 1)/*m*!	496	496	5456
Q (standard symbols)	11330.14	23265.9	73637.9
Q (equivalent symbols)	10778.34	22821.98	58943.23
Degrees of freedom (standard symbols)	960	960	29790
Degrees of freedom (equivalent symbols)	495	495	5455
p-value (standard symbols)	0	0	0
p-value (equivalent symbols)	0	0	0
Significance level	0.05	0.05	0.05

For Group 1 and 2, the degree of freedom for the chi-squared test was 495 and the critical value was 548.92 at the significance level of 0.05. For Group 3, the degree of freedom was 5455 and the critical value was 5627.94. The observed Q statistics were 10778.34, 22821.98, and 58943.23, respectively, all far beyond the critical values.

The three groups of experimental results denied the assumption of spatial independence, indicating that the manufacturing firms in Shenzhen were not spatially independent in distribution. Therefore, the manufacturing industry in Shenzhen followed a certain distribution pattern in space, which will be discussed later. This study focused only on the combinations of industrial divisions for manufacturing firms that are frequently located together in Shenzhen, that is, the co-location pattern. Since the order of firm divisions in the co-location pattern is not relevant, we only use equivalent symbols (as explained at the beginning of the method section) for the analysis of the unordered pattern in this study. To obtain significantly frequent equivalent symbols, the expected relative frequency and 95% confidence interval for each symbol under the independent assumptions were calculated in this study. Equivalent symbols with observed relative frequency greater than the expectation and beyond the 95% confidence interval were identified as significantly frequent equivalent symbols.

When *m* = 2, the equivalent symbols consisted of a central point and its nearest neighbor. When *r* = 0, the observed frequencies of 220 equivalent symbols were significantly greater than the expected values. When *r* = 1, the observed frequencies of 228 equivalent symbols were significantly greater than the expected relative frequency values, identifying pairs contributing to the co-location phenomenon.

Symbols with high observed frequency indicate frequently occurring co-location patterns, highlighting prominent agglomerations of industrial divisions. Analyzing equivalent symbols with high observed frequency is crucial for informing the site planning of various manufacturing divisions. Moreover, such prominent symbols are also less susceptible to noise interference compared to low-frequency ones. The following sections will focus on these high-frequency symbols.

**Fig 4:**
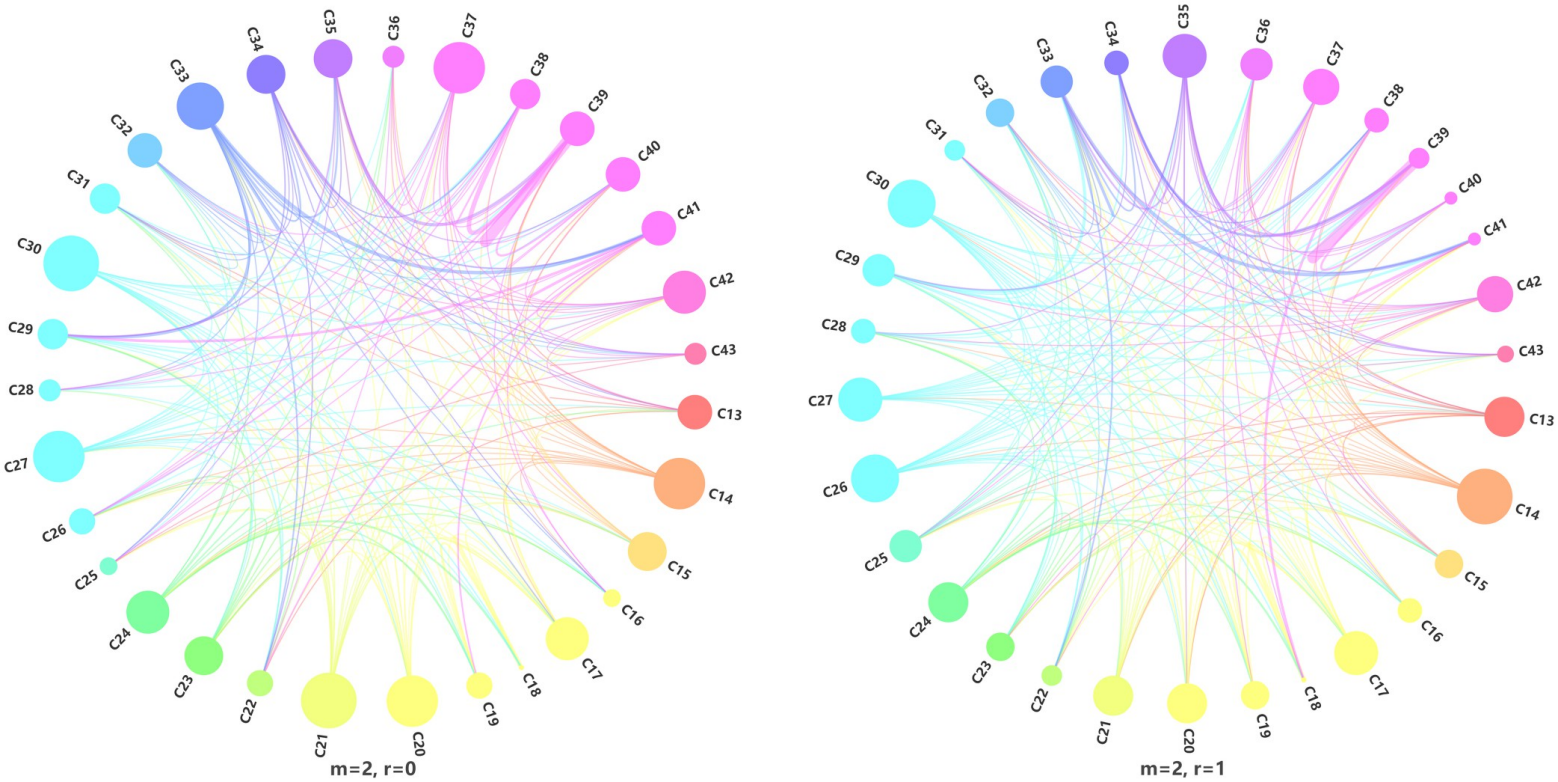
String plot of the manufacturing division pairs representing frequently co-located firms.

Fig 4 illustrates co-location relationships between different divisions of manufacturing firms, where observed frequencies exceed the expected relative frequencies in independent processes and other manufacturing industries when *m* = 2 and *r* equals 0 or 1. The size of each circle corresponds to the number of other manufacturing divisions associated with the division it represents, while the thickness of the arcs between circles indicates the observed frequency of the division pair represented by the equivalent symbols.

The division of Other Manufacturing (C41) is excluded from the following analysis because it does not belong to any of the categories listed in Table 1. Five patterns with the same divisions (C39-C39, C18-C18, C33-C33, C29-C29, and C38-C38) and nine patterns with different divisions (C35-C39, C29-C33, C39-C40, C18-C19, C18-C24, C33-C35, C33-C34, C22-C33, and C26-C39) were observed (Fig 4). These pairs were also further validated with the kernel density method in S2 Appendix.

When *m* = 3 and *r* = 2, the equivalent symbols consist of three elements, known as 3-tuple. A total of 5456 triplets were identified in this study. It was found that most of these 3-tuples comprised mainly high-frequency divisions of manufacturing (Table 3). As noted earlier, though the division of other manufacturing was denoted by C41, this category was not included in further analysis due to its low frequency and limited significance.

**Table 3:** Manufacturing divisions with frequent appearance in identified triplets.

Manufacturing division name	Proportion
C39–Manufacture of computer, communication and other electronic equipment	26.19%
C18–Manufacture of wearing apparels (except fur apparels)	11.21%
C33–Manufacture of fabricated metal products	10.77%
C29–Manufacture of rubber and plastic products	7.23%
C38–Manufacture of electrical machinery and equipment	6.47%
C24–Manufacture of educational, artistic, sports and recreational items	3.81%
C35–Manufacture of special-purpose machinery and equipment	3.68%

Equivalent symbols for each category listed in Table 3 were filtered, from which the symbols with high observed frequency were selected. We focus on these selected symbols as representative types of co-location patterns for further analysis and discussion.

### Visual analysis of prominent co-location pattern

**Fig 5:**
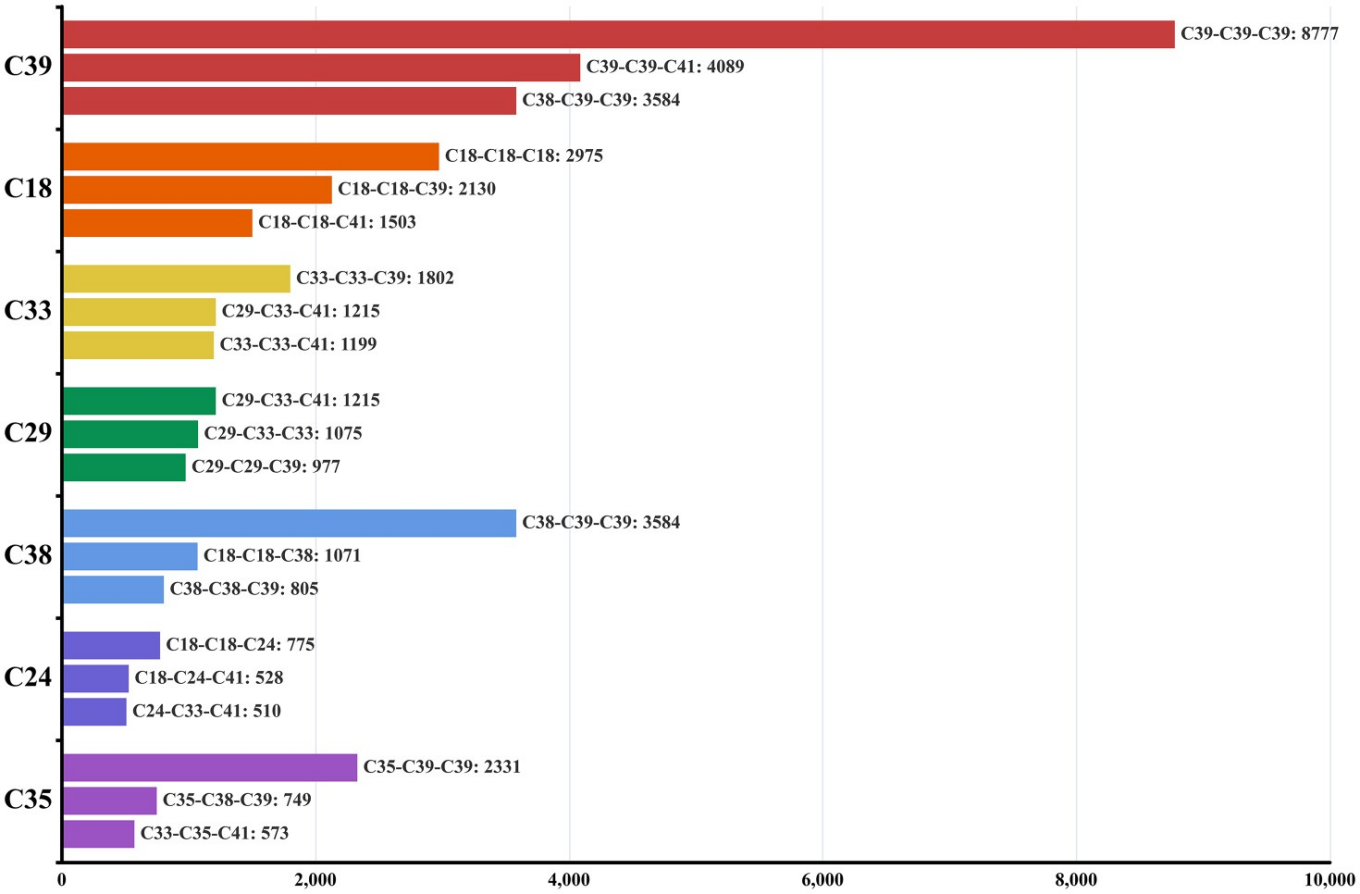
Observed frequency of top-three equivalent symbols in each of the prominent manufacturing divisions.

Meanwhile, in order to inspect the spatial distributions of typical equivalent symbols for each prominent category in Table 3 (which largely overlaps with the sectors from prominent 2-tuples), only equivalent symbols with the top three frequencies in the category are plotted in Fig 5. We use the center points of 3-surroundings that correspond to these selected equivalent symbols to represent the location of an instance. Three equivalent symbols in each map are differentiated by three levels of color intensity of the point markers.

**Fig 6:**
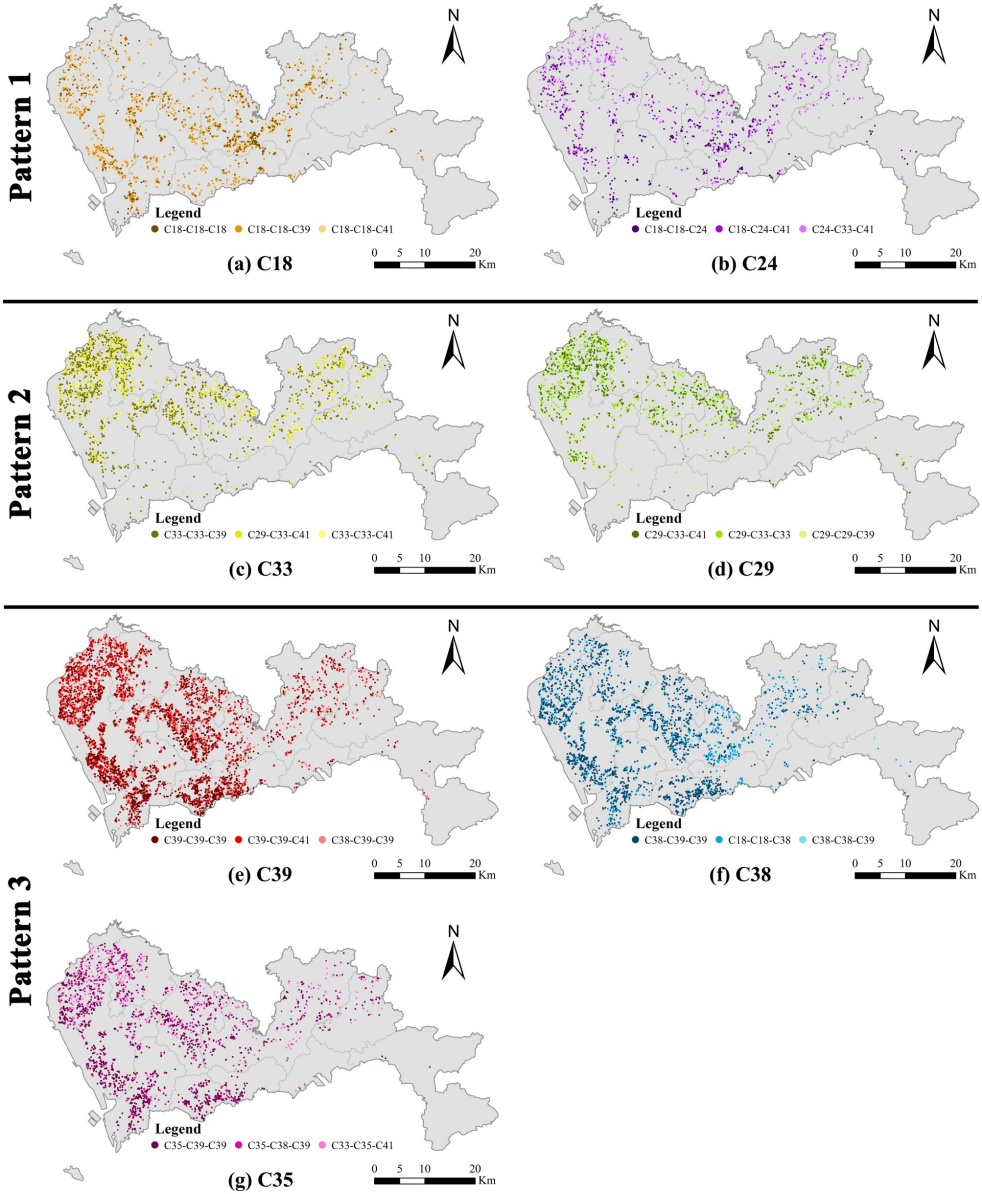
Spatial distribution of instances of frequent equivalent symbol pairs and triplets. Each group of pattern represent a typical characteristic of firms, including labor-intensive, intermediate-demand, and technology-intensive, as defined in the data section. (Source: The authors).

Fig 6 shows that the clusters of wearing apparels (except fur apparels) industry (C18) and educational, artistic, sports and recreational items manufacturing industry (C24) were principally located in the middle, middle-north, and southwest regions of Shenzhen. The clusters of the fabricated metal products industry (C33) and rubber and plastic products manufacturing industry (C29) were principally located in the northwest and middle-northwest regions of Shenzhen, and were also scattered in the northeast region. The intra-industry and inter-industry clusters of computer, communication, and other electronic equipment manufacturing industry (C39), electrical machinery and equipment manufacturing industry (C38), and special-purpose machinery and equipment manufacturing industry (C35) were principally located in the northwest, southwest, and middle-west regions of Shenzhen.

From the maps of each prominent manufacturing sector in Fig 6, we further consider the spatial interaction of those firms using additive color mixing technique.

**Fig 7:**
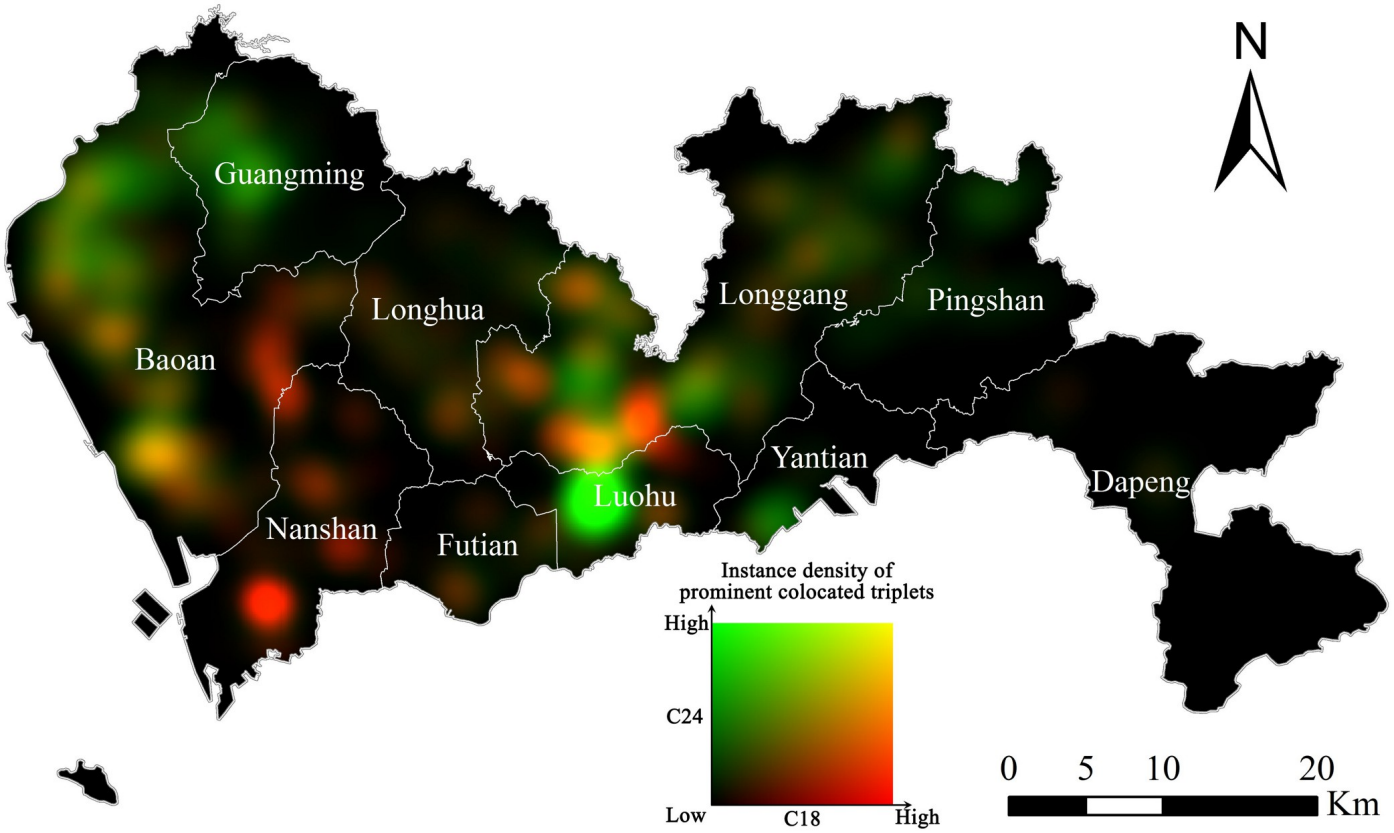
Additive color mixing visualization of C18 and C24 firms. The density of prominent triplets involving C18 firms is represented by tones of red while that of triplets involving C24 firms corresponds to tones of green. (Source: The authors).

Fig 7 illustrates the colocation of firms manufacturing wearing apparels (C18) and educational and sports items (C24), with red tone representing density of C18 firms and green tone for C24. It can be seen that the agglomeration of C18 firms (reddish areas) are located in Nanshan and east part of Bao’an; the agglomeration of C24 firms (greenish areas) are located in Guangming, Luohu, and northwest of Bao’an; the colocation, or mixing, of C18 and C24 firms (yellowish areas) are located in the southwest of Bao’an and central parts of the city.

**Fig 8:**
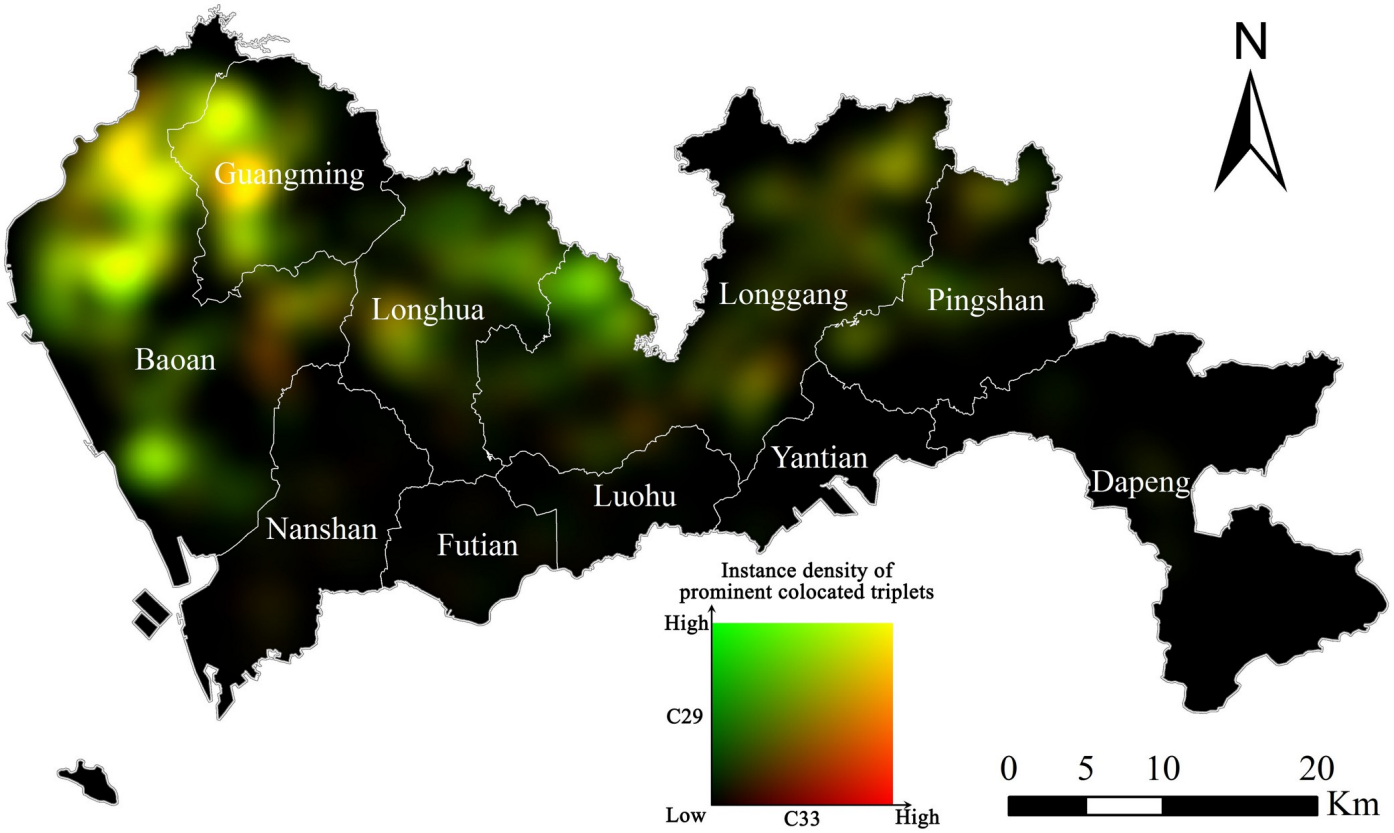
Additive color mixing visualization of C33 and C29 firms. The density of prominent triplets involving C33 firms is represented by tones of red while that of triplets involving C29 firms corresponds to tones of green. (Source: The authors).

Fig 8 illustrates the colocation of C33 (metal) and C29 (rubber and plastic) firms, with red tone representing density of C33 firms and green tone for C29. It can be seen that the agglomeration of C18 firms (reddish areas) are located in northeast of Bao’an and southwest of Longhua; the agglomeration of C29 firms (greenish areas) are located in the south part of Bao’an, northeast of Longhua, and west of Longgang; the colocation of C33 and C29 firms (yellowish areas) are located in the northeast of Bao’an, west of Guangming, east of Longgang, and northwest of Pingshan.

**Fig 9:**
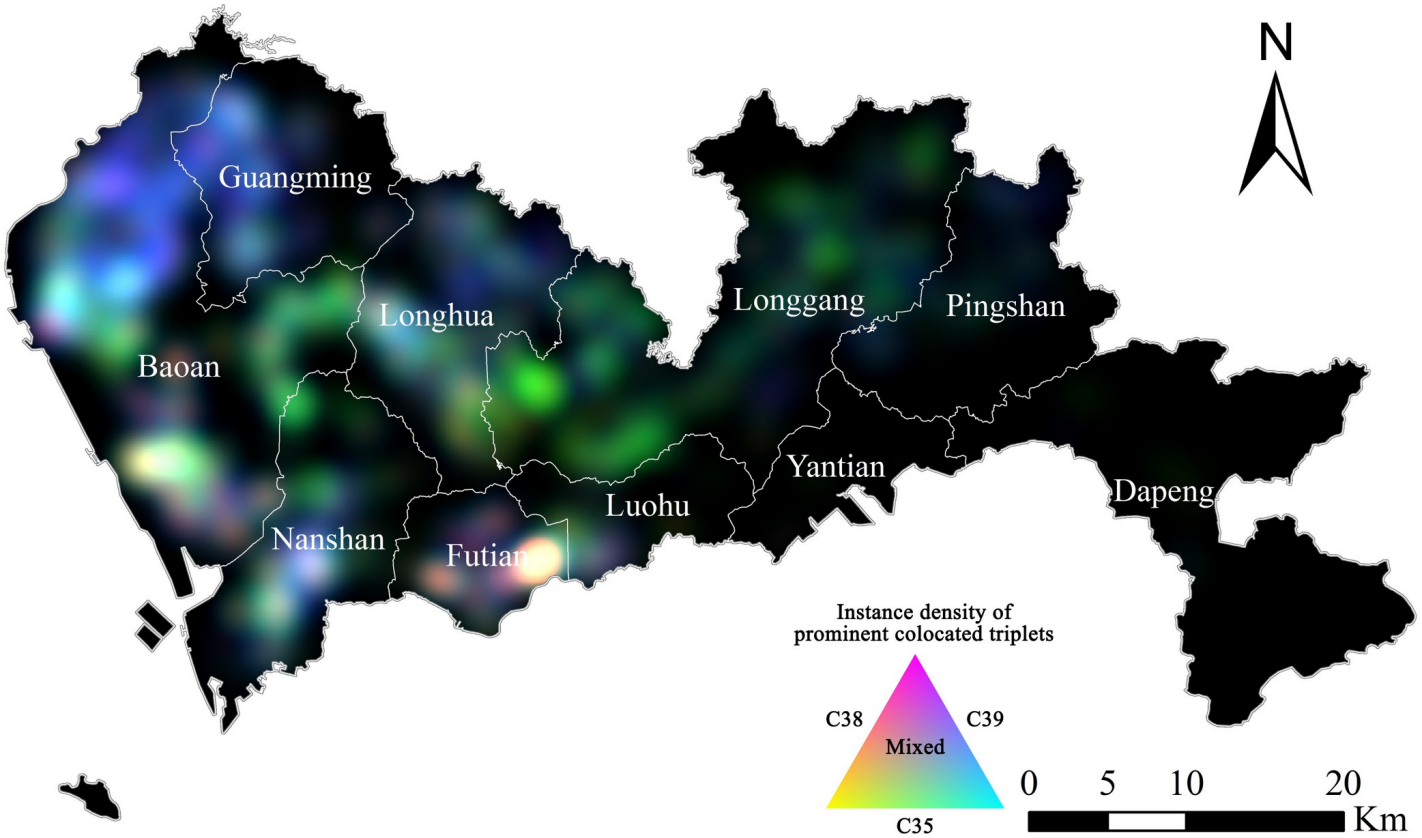
Additive color mixing visualization of C39, C38 and C35 firms. The density of prominent triplets involving C38 firms is represented by tones of red; density of triplets involving C35 firms corresponds to tones of green; density of triplets involving C39 firms corresponds to tones of blue. Therefore, magenta indicates mixing of prominent triplets involving C39 and C38 firms; yellow indicates mixed instances of C38 triplets and C35 triplets; cyan indicates mixed instances of C39 triplets and C38 triplets. (Source: The authors).

Fig 9 illustrates the colocation of firms manufacturing computers (C39, red), electronic and machinery (C38, green), and special equipment (C35, blue). For colocation within each sector, there are few instances for the agglomeration of C39 firms (reddish areas). The agglomeration of C38 firms (greenish areas) are located in the east of Bao’an, west and northeast of Longgang. The agglomeration of C35 firms (blueish areas) are located in the north of Bao’an and west of Guangming. For colocation involving two sectors, the pairs of C39 and C38 firms (yellowish areas) are rarely observed; the pairs of C38 and C35 firms (cyan areas) are located in the northwest of Bao’an and west of Guangming; the pairs of C39 and C35 firms (magenta areas) are located in central Nanshan and Futian. For the colocation of all three sectors, the whitish areas are located in the south of Bao’an and the east of Futian.

## Discussion and implication

### Driving factors of colocation patterns

Significant co-located pairs and triplets of manufacturing divisions were detected using Q statistics in this paper, revealing prominent cases of agglomeration. Industrial agglomeration is a dynamic process coordinated by different firms from both industrial and spatial aspects; the driving mechanisms behind are diverse and worth exploring [53]. With regard to the major co-location patterns found, the agglomeration patterns and their driving factors of labor-intensive, intermediate-demand, and technology-intensive manufacturing firms are characterized and summarized with reference to Marshall’s externalities [7].

Labor-intensive firms are mainly co-located with others in the same division, showing specialization characteristic. For example, C18 firms were observed to appear in co-location patterns C18-C18 and C18-C18-C18 within the same division, as well as the co-location patterns C18-C24 and C18-C18-C24 across divisions. Amidst China’s diminishing demographic dividend, labor-intensive firms are now facing labor shortages and rising labor costs [54, 55]. In this scenario, the advantages of labor pooling have become more pronounced, potentially emerging as the predominant incentive for these firms to cluster [56].

Intermediate-demand firms also co-locate across divisions by establishing connections with downstream firms in the industry chain. For instance, C33 was identified in equivalent symbols such as C33-C33 and C33-C34 as frequent pairs, along with C33-C33-C39 as frequent triplets. This observation suggests that C33 may serve as an upstream supplier by providing raw materials or essential components for the production of C34 and C39. The vertical input-output relationship induces intermediate-demand firms to agglomerate around final-demand firms, typically leading to an expanded market size and an increase in the division of labor—a strategic orientation for cluster development [57].

Technology-intensive firms tend to form intra-division agglomerations, especially specialized ones. The distinctive agglomeration around C39 is evidenced by reflexive co-location patterns like C39-C39 and C39-C39-C39, as well as co-location patterns with firms from another manufacturing division within relevant domains, such as C39-C39-C41 and C38-C39-C39. These specialized agglomerations play a pivotal role in catalyzing technological innovation by facilitating communication. Simultaneously, they foster internal competition, accelerating the pace of innovation and product iteration, as a manifestation of Porter externalities [58, 59]. Co-location patterns like C35-C38-C39 were observed in diversified agglomerations of firms from different technological domains, providing a potential avenue for knowledge reorganization to promote radical innovation, as a manifestation of Jacobs externalities [20, 60]. Under either interpretation of externalities, the observed results underscore the emphasis on the role of knowledge spillovers. As the driving forces of economic growth shift from factor investment to innovation, the significance of knowledge spillovers becomes increasingly important in Shenzhen, where innovation is becoming the dominant development strategy [61], and may outweigh the benefits of labor pooling, input sharing, and transportation cost reduction [62–64].

### Spatial coupling of industrial agglomerations

The additive color mixing visualization reveals agglomeration or co-location among manufacturing divisions, as well as a converging tendency in the site selection preferences of firms. To promote coordinated industrial development and avoid losses caused by spatial mismatch, it is beneficial to discuss the spatial coupling among agglomerations of firms from different divisions.

Co-location patterns related to labor-intensive firms often agglomerate at specific points, while the locations of these agglomerations are relatively scattered [7, 65]. Instances of co-location between C18 and C24 firms are spatially separated, forming significant but less coupled agglomerations, matching the spatial pattern of retail locations identified by Wang et al. [66]. This suggests that retailing might be indicative of final-demand manufacturing agglomeration [67]. Agglomerations related to labor-intensive firms, especially C18 firms, basically overlap with agglomerations of technology-intensive firms, consistent with the findings of Ye et al. [68]. Given that the trend of smart manufacturing is creating opportunities for labor-intensive firms to reduce costs [69], these firms may situate themselves close to technology-intensive agglomerations to empower their transition and upgrade to smart manufacturing.

Co-location patterns related to intermediate-demand firms demonstrate strong spatial continuity, with multiple interconnected agglomerations forming corridors, such as C33 and C29 firms. There is a significant overlap between the agglomerations of intermediate-demand firms and technology-intensive firms, fostering coordination between upstream and downstream firms. However, this trend of agglomeration was not observed in the downtown areas, where technology-intensive firms are also clustered. This discrepancy could be attributed to the scarcity of raw materials and resources in these downtown areas. Intermediate-demand firms, relying heavily on raw material inputs, may consider spatial distance as a crucial factor in their location decisions [70].

Co-location patterns related to technology-intensive firms span vast areas. Although the co-location phenomena involving C35, C38, and C39 firms are not entirely coupled, there is a converging trend in areas with clusters of high-tech firms like C35 as suggested by the study of Yu et al. [61]. This convergence points to the central position of C35 as a probable source of knowledge spillover within the co-location of C35, C38, and C39 firms. Agglomeration involving technology-intensive firms significantly overlaps with intermediate-demand firms, facilitating the creation of clusters where large firms at the core collaborate and compete with each other. Simultaneously, small and medium-sized firms consistently cater to their production needs. This dynamic addresses the challenges associated with low product variety in clusters dominated solely by a few large firms, ultimately enhancing overall competitiveness and adaptability to environmental changes [71].

### Implications

The collaboration among clustered firms is essential for enhancing industrial agglomerations. Firstly, our findings reveal that industrial agglomeration predominantly occurs across specialized divisions, including labor-intensive, intermediate-demand, and technology-intensive divisions, leading to the formation of spatially distinct clusters. This trend is observed in globally renowned specialized industrial clusters, such as the Third Italy in north-central Italy, Silicon Valley and Massachusetts Route 128, and Zhongguancun in Beijing. Drawing from the experiences of these clusters, implementing a unified approach to agglomeration operation and branding is crucial for enhancing product sales and reducing costs by influencing consumer behavior [72, 73]. Firms within the agglomeration should collaboratively adopt a shared branding strategy and identity, leveraging joint marketing initiatives, including advertising, promotions, and publicity campaigns, to achieve greater visibility and external economies of scale.

Secondly, cooperation models must be customized to align with the prevailing relationships among cluster firms. Our study highlights the significance of technology-intensive firms, such as C35, as crucial catalysts in agglomeration, attracting firms from both within and across divisions. For these firms, fostering knowledge sharing, technology collaboration, and research and development partnerships with counterparts is essential to continuously enhance products and services, thereby maintaining competitive advantages. Simultaneously, establishing efficient supply chains with upstream industries ensures stability and quality in raw material supply while curtailing production costs. By connecting local firms through established production and innovation networks, spillover effects are strengthened, ultimately facilitating cluster-wide upgrades.

Thirdly, the essence of cooperation lies in resource interaction, and the increase in resource mobility can be seen as an underlying catalyst. Based on our results, we speculate that technology spillovers may be the essential driver of agglomeration, particularly for technology-intensive firms. Given the growing significance of information resources in the digital economy [74], serving as an irreplaceable medium for technological spillovers, enterprises can invest in digital infrastructure development, including data centers and cloud computing, to facilitate information integration by optimizing information management and sharing. Once geographical barriers for information are lifted, firms can seek better solutions through cross-regional cooperation [75] and optimizing the allocation of resources.

## Conclusion

This study employs Q statistics (adapted with kd-tree), and additive color mixing techniques to identify and interpret co-location patterns from extensive spatial point data of manufacturing firms. While we identify frequent pairs and triplets of manufacturing divisions, our investigation focuses on seven major divisions representing three typical production characteristics. Despite the extensive sample size, this study analyzes only the most frequent patterns, highlighting a limitation of the Q test for datasets with numerous categories. In such cases, the test may identify a large number of co-location modes, aligning with the perspective of Liu et al. [27]. The insights from these predominant patterns can guide the management and upgrading of relevant firms and agglomerations. However, it’s important to note that numerous firms and agglomerations may exhibit less prominent patterns, necessitating further examination.

Since the method used in this study, i.e. Q statistic and additive color mixing, focuses on the statistical significance and visual analytic of colocation patterns, the driving factors of the observed colocation patterns remain on a speculative basis and are beyond the capability of the approach. The mechanism behind the agglomeration is still worth investigation and modelling in future work. Additionally, the analysis is set in the scope of a city, which ignores interactions of firms across the municipal boundary at a regional level. This means the findings could reflect more on the microscopic interaction within industrial campuses or agglomerations. The points in the discussion should be approached and interpreted with caution for practical use.

## Supporting information

S1 Appendix Maths of Q statistic. (PDF)

S2 Appendix Kernel density cross validation. (PDF)

S3 Appendix Computation performance. (PDF)
