# Supplementary material for: Mining co-location patterns of manufacturing firms using Q statistic and additive color mixing
Source: PLoS One. 2024 Mar 6;19(3):e0299046. doi: 10.1371/journal.pone.0299046 (PMC10917271; doi:10.1371/journal.pone.0299046)
Supplement: S1 Appendix — (PDF) [file pone.0299046.s001.pdf]

S1 Appendix. Maths of Q statistic for Mining co-location patterns of manufacturing firms using Q statistic and additive color mixing

The Q statistic is defined as the entropy of symbols while the entropy is further based on the frequency of symbols. For N locations and m surroundings with r overlapping degree, the relative frequency p of a symbol σ_j is defined as $p_{\sigma_j} = \frac{n_{\sigma_j}}{[\frac{N-m}{m-r}]+1}$. Given this frequency distribution, the entropy $h(m)$ of observed symbols is $\sum_j p_{\sigma_j} \ln p_{\sigma_j}$. Then, the Q statistic is represented as $2([\frac{N-m}{m-r}]+1)(\eta - h(m))$ where η is the upper bound of $h(m)$ given by the possible number of types k and size of surrounding m.
