# Supplementary material for: Mining co-location patterns of manufacturing firms using Q statistic and additive color mixing
Source: PLoS One. 2024 Mar 6;19(3):e0299046. doi: 10.1371/journal.pone.0299046 (PMC10917271; doi:10.1371/journal.pone.0299046)
Supplement: S2 Appendix — (PDF) [file pone.0299046.s002.pdf]

S2 Appendix. Kernel density cross validation for Mining co-location patterns of manufacturing firms using Q statistic and additive color mixing

For the prominent pairs identified by the Q statistic, the colocation patterns were also validated using the kernel density method which is more familiar to the economic community. The Gaussian kernel was used with a bandwidth of 4.5km to guarantee at least one neighboring location for each division of manufacturing industry. Since the kernel density algorithm is not designed or adapted for large datasets, only 10% of the data were sampled in a stratified manner from the population so that the memory footprint for the computation was acceptable. The observed point density curve and a group of upper and lower bounds are plotted in Figure A. All of the fourteen pairs exhibit clustering trend for at least one distance interval below 35km. There are also seven pairs of manufacturing divisions with another significant clustering distance range above 35km.

The results of colocation pairs are consistent with those detected by the Q statistic. This reflects the robustness of Q statistic approach in case of point patterns with

insignificant clustering or even repelling distance intervals for large datasets. The envelop curves of the kernel density method enables a multiscale analysis and the detailed information of the characteristic distance intervals facilitate the understanding of the colocation pairs. However, it is only applicable to the analysis of pairs while the Q statistic has the capability of identify triplets and even longer patterns of point types.

Figure A: **Observed and envelop kernel density of frequently co-located pairs of manufacturing divisions.** See next page.

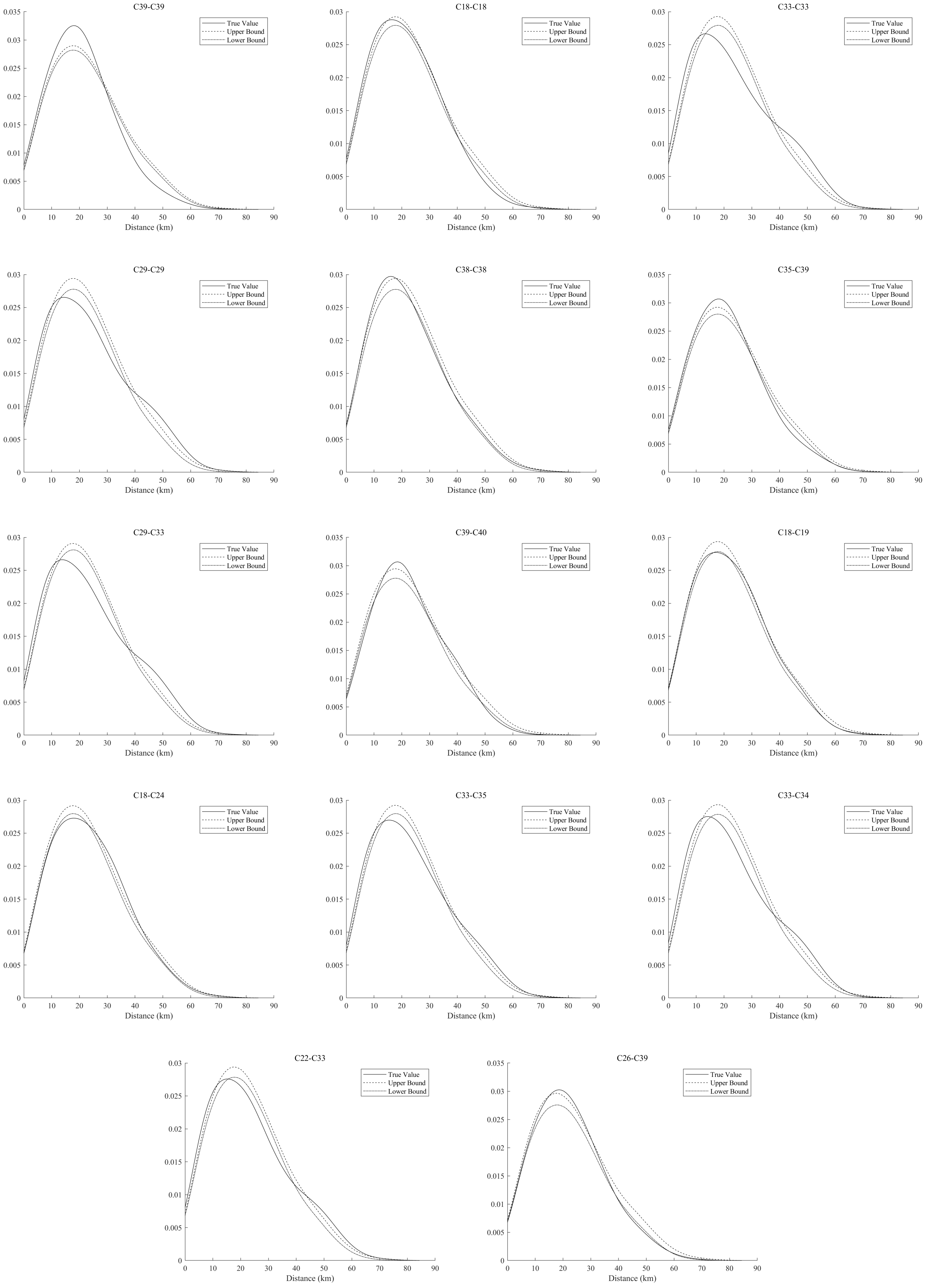
C39-C39
C18-C18
C33-C33
C29-C29
C38-C38
C35-C39
C29-C33
C39-C40
C18-C19
C18-C24
C33-C35
C33-C34
C22-C33
C26-C39
True Value
Upper Bound
Lower Bound
Distance (km)
