# Supplementary material for: Mining co-location patterns of manufacturing firms using Q statistic and additive color mixing
Source: PLoS One. 2024 Mar 6;19(3):e0299046. doi: 10.1371/journal.pone.0299046 (PMC10917271; doi:10.1371/journal.pone.0299046)
Supplement: S3 Appendix — (PDF) [file pone.0299046.s003.pdf]

S3 Appendix. Computation performance for Mining co-location patterns of manufacturing firms using Q statistic and additive color mixing

To demonstrate the contribution of the proposed method using Kd-tree, the computation cost for a series of data sizes were measured and the reduction was validated. The specific computing platform used for the implementation and tests was a laptop computer with i7-13700F CPU at 2.10 GHz, 64GB memory, running Windows 10 operating system and Matlab 2022.

The technical improvement is two-fold, including time and memory space. According to Table B, the introduction of kd-tree could reduce the time when the number of data points is over 10k points, and the improvement increases rapidly to 56% with 50k points. Memory usage improvement started from 2k points, and it is reduced even more by 99% with 50k points, indicating that the proposed method could support larger datasets given the same amount of available memory.

Table B: **Computation cost reduction.**

Indicator	Number of points	1000	2000	5000	10000	20000	50000
Time/s	Previous	0.038	0.094	0.347	1.067	5.283	29.786
	Proposed	0.076	0.166	0.470	1.162	4.442	12.896
	Improvement	-100%	-76.4%	-35.7%	-9.0%	15.9%	56.7%
Memory/KB	Previous	656	8840	10208	12376	22728	35711056
	Proposed	3280	5868	6480	6968	12696	27724
	Improvement	-400%	33.6%	36.5%	43.7%	44.1%	99.9%
